# The Association Between Spicy Food Intake and Risk of Hyperuricemia Among Chinese Adults

**DOI:** 10.3389/fpubh.2022.919347

**Published:** 2022-07-06

**Authors:** Qinwen Luo, Rui Ding, Liling Chen, Xiaoqing Bu, Meng Xiao, Xiang Liu, Yunyun Wu, Jingru Xu, Wenge Tang, Jingfu Qiu, Xianbin Ding, Xiaojun Tang

**Affiliations:** ^1^School of Public Health, Research Center for Medicine and Social Development, Chongqing Medical University, Chongqing, China; ^2^First Clinical Medical College, Chongqing Medical University, Chongqing, China; ^3^Chongqing Center for Disease Control and Prevention, Infectious Disease Control and Prevention Institute, Chongqing, China; ^4^Department of Epidemiology and Health Statistics, West China School of Public Health, Sichuan University, Chengdu, China

**Keywords:** spicy food intake, hyperuricemia, gender difference, age difference, adults

## Abstract

Hyperuricemia is associated with substantial health and economic burden all over the world. Dietary habits are an important influencing factor of hyperuricemia. This study aimed to investigate the relationship between spicy food intake and hyperuricemia based on a large population. A total of 22,125 individuals aged 30–79 were enrolled in China Multi-Ethnic Cohort (CMEC), Chongqing region. Spicy food intake information was collected by a standardized questionnaire. The association between spicy food intake and hyperuricemia was estimated by multivariable logistic regression models and multiple linear regression models. Additionally, we explored these relations in subgroups stratified by sex and age. Furthermore, sensitivity analyses were conducted to verify the stability of current findings. After controlling for potential confounders, compared with participants who never consumed spicy food and consumed less hot, participants who ate 3–5 days per week and very hot had the highest risk of hyperuricemia; the ORs (95% CIs) were 1.28 (1.09, 1.5) and 1.22 (0.92, 1.63), respectively. Additionally, the corresponding ORs (95% CIs) for each level increment in the frequency and degree of pungency in spicy food intake were 1.04 (1.01, 1.07) (*P* trend = 0.009) and 1.15 (1.04, 1.26) (*P* trend = 0.004). Further in sex-stratified and age-stratified analysis, similar positive associations were observed among men and those aged 30–59, but no significant association was found among women and those aged 60–79. In the linear regression models, 3–5 days per week and moderate pungency in spicy food intake were associated with 5.21 μmol/L (95% CI: 1.72, 8.70) and 4.69 μmol/L (95% CI: 1.93, 7.45) higher serum urate level. Results in further subgroup analysis were generally consistent with the logistic regression models. This study suggests that spicy food intake may be a risk factor for hyperuricemia, especially in men and younger people, and more studies are warranted to verify the causal associations.

## Introduction

Hyperuricemia is associated with substantial health and economic burden worldwide, which has attracted increasing recognition in recent years. In addition to mainly causing gout, hyperuricemia is often associated with several other diseases including chronic kidney disease, cardiovascular diseases, and metabolic syndrome (1–3). Its comorbidities also increase the risk of type 2 diabetes mellitus, hypertension, overweight and obesity, hypertriglyceridemia, and hypercholesterolemia (4). The prevalence of hyperuricemia was 14.6% in the United States and 16.6% in South Australia (5, 6). The latest data show that the overall prevalence of hyperuricemia in China is 13.3%, which has become a common metabolic disease after diabetes (7, 8). Compared with developing countries, the prevalence of hyperuricemia is generally higher in developed countries, but it has been increasing in many developing countries (9–11). With the development of the economy and society in China in the past 30 years, the eating habits of Chinese people have significantly changed. It is worth noting that dietary patterns were related to hyperuricemia. Certain foods rich in purine, alcohol, meat, seafood, a fat-rich diet, and soft drinks consumption can increase the risk of hyperuricemia (12, 13).

Spices have been an essential part of culinary cultures worldwide, with a long history in flavoring, coloring, and preserving food, as well as medicinal use (14, 15). Spiciness or pungency was regarded as one of the basic tastes in ancient Asia, especially in India and China (16, 17). It is reported that more than 30% of adults eat spicy food every day in China (18). As a mid-west city famous for its hot pot and spicy Sichuan cooking style, pepper's annual consumption per capita is up to 96.5 kg in Chongqing (19). There is increasing evidence that hyperuricemia or elevated serum uric acid (SUA) levels are associated with metabolic syndrome (MetS) and its components (20–22). Several previous studies have explored the relationship between spicy foods and metabolic diseases, indicating that the consumption of chili peppers was positively related to obesity, abnormal lipid metabolism, and insulin resistance (23–25). However, few previous studies have investigated the association between spicy food intake and hyperuricemia. Therefore, based on the China Multi-Ethnic Cohort (CMEC) data in Chongqing, the current study aimed to investigate the relation between spicy food intake and hyperuricemia.

## Methods

### Study Population

**Figure 1:**
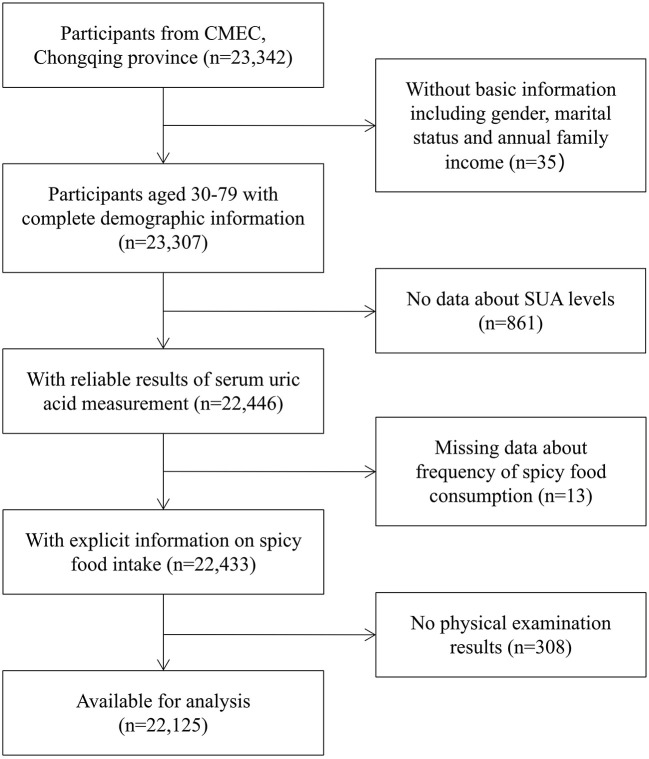
Flowchart for participants' selection.

We gathered the data from the CMEC (Chongqing region). Details of the cohort study design have been presented previously (26). All participants in our study were randomly selected from 13 districts/counties. The inclusion criteria we applied were as follows: aged 30–79 years on the day of the investigation; lived in the local area for half a year or more and capable of completing the baseline survey; voluntarily participating in the study and signing informed consent; having the normal ability of expression and understanding. Overall, 23,342 individuals aged 30–79 were recruited at baseline. To explore the relationship between spicy food intake and hyperuricemia, 1,217 subjects were excluded because of the following reasons: missing basic demographic information; missing data on the determination of the status of hyperuricemia; incomplete information on spicy food intake; and missing data on physical examination results. Ultimately, the study enrolled 22,125 individuals for the current analysis. Details of participants' selection were presented in Figure 1.

### Ascertainment of Hyperuricemia

Blood samples were collected from all participants *via* venepuncture after overnight fasting, then centrifuged and separated for later clinical laboratory testing, including fasting blood glucose (FBG), lipid profiles, and serum uric acid (SUA). The SUA was analyzed by the standard enzyme method, which was based on the generation of hydrogen peroxide from uric acid catalyzed by immobilized uricase, and then determined by the color reaction catalyzed by immobilized peroxidase. The intra-assay coefficient of variation for SUA was <6.5%. In this study, we defined hyperuricemia as increased SUA above 417 μmol/L (7 mg/dL) in men and above 357 μmol/L (6 mg/dL) in women, as described in previous studies (27, 28).

### Assessment of Spicy Food Intake

The standardized questionnaire used to collect dietary intake information was delivered by well-trained staff through face-to-face interviews. Participants were asked, “How frequently did you have spicy foods during the past month?” with the following five response categories: “never,” “ <1 d/week”, “1–2 d/week”, “3–5 d/week,” or “6–7 d/week.” Among those weekly consumers (participants who consumed spicy food at least 1 day a week), participants were further asked, “Which kind of spicy food do you prefer?” with three response categories: “less hot,” “moderately hot,” or “very hot.”

### Assessment of Covariates

A structured questionnaire was used to collect detailed information regarding sociodemographic characteristics and lifestyle factors. Besides the essential demographic characteristics including age, sex, educational level, marital status, and annual family income, the following variables that may affect the prevalence of hyperuricemia were explored in the current study: smoking status, alcohol consumption, physical activity, total energy intake, dietary pattern, body mass index (BMI), hypertension, type 2 diabetes mellitus, and dyslipidemia. All the introduced covariates were selected with reference to other studies on the association between spicy food and metabolic diseases (23–25, 29). Daily consumption of alcohol (grams of pure alcohol per day) was calculated based on the reported frequency and quantity of drinking. Participants' physical activity was converted into metabolic equivalent tasks (METs) spent on work, transportation, housework, and leisure time activities (30). The food frequency questionnaire (FFQ) contained 13 main food groups and was completed by all participants to collect their dietary intake data in the baseline survey. The average daily total energy intake (including protein, fat, and carbohydrates energy intake) for each participant was calculated based on dietary information according to the Chinese Food Composition Table 2004. The dietary pattern was estimated by the Dietary Approaches to Stop Hypertension (DASH) diet score, which focused on seven kinds of food, including fresh fruits, vegetables, beans, dairy, whole grain intake, red and processed meats, and sodium salt. Each kind of food was assigned one to five scores based on the quintile of the average food intake, and then all the food scores were added up to get an overall DASH score (31). BMI was calculated as weight divided by height squared (kg/m^2^). In the present study, hypertension was defined as having an average measured SBP/DBP ≥ 140/90 mmHg or self-reported history of hypertension diagnosed by doctors (32). Diabetes mellitus was defined as an FBG level of ≥ 7 mmol/L or a history of physician-diagnosed diabetes. Participants with any one of the following components were considered as having dyslipidemia: (1) serum total cholesterol (TC) ≥ 6.22 mmol/l; (2) triacylglycerol (TG) ≥ 2.26 mmol/l; (3) low-density lipoprotein cholesterol (LDLC) ≥ 4.14 mmol/l; (4) and high-density lipoprotein cholesterol (HDLC) < 1.04 mmol/l (33).

### Statistical Analysis

The general characteristics of study participants were described according to the spicy food intake frequency (never, <1, 1–2, 3–5, 6–7 d/week) and the degree of pungency in spicy food (low, moderate, high), respectively. Means and standard deviations were presented for continuous variables, while categorical variables were expressed as percentages. ANOVA and chi-square tests were used to compare the differences in covariates among various groups of spicy food intake. The linear trend between increasing spicy food frequency and covariates was evaluated using Spearman correlation and Cochran-Mantel-Haenszel tests for continuous and categorical variables, respectively. Multivariable-adjusted logistic regression models were conducted to investigate the association between spicy food intake frequency, degree of pungency in spicy food, and hyperuricemia. And the results of the odds ratios (ORs) and 95% CIs were presented. A series of models were used to minimize the influence of confounding factors on this association. Model 1 was a crude model without any adjustments; model 2 adjusted for age, sex, marital status, educational level, and annual family income; model 3 additionally adjusted for smoking status, alcohol consumption, physical activity, total energy intake, DASH score, BMI, hypertension, type 2 diabetes mellitus, and dyslipidemia status. Considering the prevalence of hyperuricemia in men was higher than that in women and increased with age (8, 34, 35). Additionally, we explored these relations in subgroups stratified by sex and age. The interaction tests of spicy food intake on age, sex, educational level, marital status, annual family income, BMI, smoking, alcohol drinking, physical activity, more meat, and more fish were also examined in logistic regression models. Sensitivity analyses were conducted by excluding participants with self-reported peptic ulcer disease, coronary heart disease, stroke, and cancer (*n* = 1,992) to verify the stability of our findings. All statistical analyses were carried out using SPSS version 26.0. All statistical tests were two-sided, and a *P* < 0.05 was considered statistical significance.

## Results

### General Characteristics

Table 1 presented the participants' general characteristics by five spicy food intake frequency groups. Of the 22,125 participants, the mean age was 51.4 ± 11.72 years. Compared with participants who never consumed spicy food, those who ate spicy food frequently were more likely to be younger, a man, married or cohabiting, with higher educational level, higher annual family income, have more physical activity, smokers, consume more alcohol, have higher total energy intake, higher BMI, and serum urate level, but less likely to have diabetes or hypertension. In addition, among weekly spicy food consumers, those who consumed spicy food more frequently tended to prefer the higher pungency of spicy food.

**Table 1:** Characteristics of the participants according to the frequency of spicy food intake.

**Variable**	**Total**	**Frequency of spicy food intake**					***P* trend**
		**Never**	**<1 d/week**	**1–2 d/week**	**3–5 d/week**	**6–7 d/week**	
No. participants	22,125	3,105	2,177	3,917	2,839	10,087	
Age (years, Mean ± SD)	51.40 ± 11.72	56.33 ± 12.22	52.33 ± 11.83	50.09 ± 11.27	49.36 ± 11.44	50.76 ± 11.36	<0.001
Males, *n* (%)	10,312 (46.61)	1,395 (44.93)	917 (42.12)	1,636 (41.77)	1,309 (46.11)	5,055 (50.11)	<0.001
Educational level, *n* (%)							<0.001
Illiteracy/primary school	7,194 (32.52)	1,511 (48.66)	775 (35.60)	1,040 (26.55)	611 (21.52)	3,257 (32.30)	
Junior high school	7,131 (32.23)	944 (30.40)	653 (30.00)	1,281 (32.70)	961 (33.85)	3,292 (32.64)	
High school and above	7,800 (35.25)	650 (20.93)	749 (34.41)	1,596 (40.75)	1,267 (44.63)	3,538 (35.07)	
Marital status, *n* (%)							<0.001
Married/cohabiting	19,451 (87.91)	2,642 (85.09)	1,893 (86.95)	3,419 (87.29)	2,497 (87.95)	9,000 (89.22)	
Separated/divorced/widowed/unmarried	2,674 (12.09)	463 (14.91)	284 (13.05)	498 (12.71)	342 (12.05)	1,087 (10.78)	
Annual family income, yuan (%)							<0.001
<12,000	2,452 (11.08)	615 (19.81)	297 (13.64)	361 (9.22)	226 (7.96)	953 (9.45)	
12,000–19,999	2,815 (12.72)	547 (17.62)	320 (14.70)	480 (12.25)	306 (10.78)	1,162 (11.52)	
20,000–59,999	7,629 (34.48)	1,084 (34.91)	721 (33.12)	1,354 (34.57)	992 (34.94)	3,478 (34.48)	
60,000–99,999	4,695 (21.22)	470 (15.14)	448 (20.58)	905 (23.10)	664 (23.39)	2,208 (21.89)	
>100,000	4,534 (20.49)	389 (12.53)	391 (17.96)	817 (20.86)	651 (22.93)	2,286 (22.66)	
Smoking status, *n* (%)							<0.001
Non-smoker	16,223 (73.32)	2,534 (81.61)	1,771 (81.35)	3,071 (78.40)	2,073 (73.02)	6,774 (67.16)	
Current smoker	4,534 (20.49)	395 (12.72)	284 (13.05)	638 (16.29)	586 (20.64)	2,631 (26.08)	
Ex-smoker	1,368 (6.18)	176 (5.67)	122 (5.60)	208 (5.31)	180 (6.34)	682 (6.76)	
Alcohol consumption (g/week, Mean ± SD)	25.42 ± 77.66	12.36 ± 57.65	14.62 ± 55.84	16.84 ± 59.17	21.99 ± 67.21	36.08 ± 93.17	<0.001
Physical activity (Mets/day, Mean ± SD)	23.95 ± 16.33	23.39 ± 17.79	23.60 ± 16.90	23.45 ± 15.39	23.13 ± 15.01	24.61 ± 16.43	0.015
DASH score, Mean ± SD	20.89 ± 4.58	19.32 ± 4.86	21.03 ± 4.71	21.70 ± 4.47	21.83 ± 4.23	20.76 ± 4.45	<0.001
Degree of pungency in spicy food, *n* (%)							<0.001
Low	13,636 (80.96)	NA	NA	3,538 (90.32)	2,390 (84.18)	7,708 (76.42)	
Moderate	2,819 (16.74)	NA	NA	340 (8.68)	411 (14.48)	2,068 (20.50)	
High	388 (2.30)	NA	NA	39 (1.00)	38 (1.34)	311 (3.08)	
BMI (kg/m^2^, Mean ± SD)	24.65 ± 3.22	24.54 ± 3.16	24.43 ± 3.17	24.39 ± 3.16	24.56 ± 3.15	24.86 ± 3.28	<0.001
Total energy intake (kcal/d, Mean ± SD)	1,878.17 ± 623.27	1,732.32 ± 604.98	1,764.92 ± 598.19	1,825.54 ± 602.69	1,876.32 ± 595.28	1,968.46 ± 634.82	<0.001
Meat consumption (g/week, Mean ± SD)	714.57 ± 622.85	611.71 ± 577.24	640.08 ± 566.95	657.07 ± 579.79	716.36 ± 601.54	784.12 ± 660.43	<0.001
Fish consumption (g/week, Mean ± SD)	418.42 ± 1,020.96	269.34 ± 833.63	355.57 ± 936.19	402.67 ± 729.29	454.83 ± 756.56	473.73 ± 1,226.67	<0.001
Type 2 diabetes mellitus, *n* (%)	2,138 (9.66)	350 (11.27)	220 (10.11)	353 (9.01)	247 (8.70)	968 (9.60)	0.020
Hypertension, *n* (%)	7,749 (35.02)	1,317 (42.42)	764 (35.09)	1,189 (30.35)	887 (31.24)	3,592 (35.61)	<0.001
Dyslipidemia, *n* (%)	5,656 (25.56)	752 (24.22)	555 (25.49)	991 (25.30)	757 (26.66)	2,601 (25.79)	0.092

*SD, Standard deviation; DASH, dietary approaches to stop hypertension; BMI, body mass index; NA, not applicable*.

### Association of Spicy Food Intake With Hyperuricemia

Table 2 revealed a positive relationship between spicy food intake frequency and hyperuricemia. After progressively controlling for potential confounders, compared with those who never consumed spicy food, the OR (95% CIs) of <1, 1–2, 3–5, and 6–7 d/week were 0.99 (0.83, 1.18), 1.03 (0.89, 1.2), 1.28 (1.09, 1.5), and 1.13 (0.99, 1.29), respectively (*P* trend = 0.009). The multilevel-adjusted analyses stratified by sex were further performed, and results showed that men who consumed spicy food <1, 1–2, 3–5, and 6–7 d/week were 1.02, 1.06, 1.44, and 1.23 times more likely to have hyperuricemia (*P* trend = 0.003). In contrast, this relationship was significantly attenuated among women (all *P* trend > 0.05). Moreover, a significant interaction between sex and frequency of spicy food consumption was found (*P*_interaction_ = 0.004) (Supplementary Table S1). In addition, when the association was explored in a stratified analysis according to age, we found that among participants aged 30–59, the adjusted OR (95% CIs) of <1, 1–2, 3–5, and 6–7 d/week were 1.01 (0.8, 1.27), 1.08 (0.88, 1.31), 1.4 (1.14, 1.72), and 1.2 (1.01, 1.43) (*P* trend = 0.005). But no significant relationship was found among those aged 60–79 (all *P* trend > 0.05). There was no significant interaction between age and spicy food intake frequency (*P*_interaction_ = 0.133) (Supplementary Table S1).

**Table 2:** Multivariable-adjusted associations (ORs and 95% CIs) between frequency of spicy food consumption and risk of hyperuricemia (*N* = 22,125).

	**Frequency of spicy food intake**					**Each level increment**	***P* trend**
	**Never (*n* = 3,105)**	**<1 d/week (*n* = 2,177)**	**1–2 d/week (*n* = 3,917)**	**3–5 d/week (*n* = 2,839)**	**6–7 d/week (*n* = 10,087)**		
**Total**							
No. events (%)	403 (12.98)	287 (13.18)	521 (13.30)	478 (16.84)	1,629 (16.15)		
Model 1	1.00 (Ref)	1.02 (0.87, 1.20)	1.03 (0.90, 1.18)	1.36 (1.18, 1.57)	1.29 (1.15, 1.45)	1.08 (1.05, 1.11)	<0.001
Model 2	1.00 (Ref)	1.02 (0.86, 1.20)	1.02 (0.88, 1.17)	1.28 (1.11, 1.49)	1.20 (1.06, 1.36)	1.06 (1.03, 1.08)	<0.001
Model 3	1.00 (Ref)	0.99 (0.83, 1.18)	1.03 (0.89, 1.20)	1.28 (1.09, 1.50)	1.13 (0.99, 1.29)	1.04 (1.01, 1.07)	0.009
**Males**							
No. events (%)	223 (15.99)	165 (17.99)	316 (19.32)	331 (25.29)	1,139 (22.53)		
Model 1	1.00 (Ref)	1.15 (0.92, 1.44)	1.26 (1.04, 1.52)	1.78 (1.47, 2.15)	1.53 (1.31, 1.79)	1.11 (1.07, 1.15)	<0.001
Model 4	1.00 (Ref)	1.02 (0.82, 1.28)	1.06 (0.87, 1.28)	1.44 (1.18, 1.74)	1.29 (1.10, 1.52)	1.08 (1.04, 1.11)	<0.001
Model 5	1.00 (Ref)	1.02 (0.80, 1.29)	1.06 (0.86, 1.31)	1.44 (1.17, 1.78)	1.23 (1.03, 1.46)	1.06 (1.02, 1.10)	0.003
**Females**							
No. events (%)	180 (10.53)	122 (9.68)	205 (8.99)	147 (9.61)	490 (9.74)		
Model 1	1.00 (Ref)	0.91 (0.72, 1.16)	0.84 (0.68, 1.04)	0.90 (0.72, 1.14)	0.92 (0.77, 1.10)	0.99 (0.95, 1.03)	0.604
Model 4	1.00 (Ref)	1.05 (0.82, 1.34)	1.03 (0.83, 1.28)	1.12 (0.89, 1.43)	1.10 (0.91, 1.32)	1.02 (0.98, 1.07)	0.258
Model 5	1.00 (Ref)	0.96 (0.74, 1.25)	1.01 (0.80, 1.28)	1.07 (0.84, 1.38)	1.03 (0.84, 1.25)	1.01 (0.97, 1.06)	0.624
**30–59 years old**							
No. events (%)	212 (11.90)	184 (11.92)	386 (12.63)	371 (16.58)	1,228 (15.95)		
Model 1	1.00 (Ref)	1.00 (0.81, 1.24)	1.07 (0.90, 1.28)	1.47 (1.23, 1.77)	1.41 (1.20, 1.64)	1.11 (1.07, 1.14)	<0.001
Model 6	1.00 (Ref)	0.99 (0.80, 1.23)	1.04 (0.86, 1.25)	1.34 (1.11, 1.62)	1.24 (1.06, 1.46)	1.07 (1.03, 1.11)	<0.001
Model 7	1.00 (Ref)	1.01 (0.80, 1.27)	1.08 (0.88, 1.31)	1.40 (1.14, 1.72)	1.20 (1.01, 1.43)	1.05 (1.02, 1.09)	0.005
**60–79 years old**							
No. events (%)	191 (14.44)	103 (16.27)	135 (15.70)	107 (17.80)	401 (16.79)		
Model 1	1.00 (Ref)	1.15 (0.89, 1.50)	1.10 (0.87, 1.40)	1.28 (0.99, 1.66)	1.20 (0.99, 1.44)	1.04 (1.00, 1.09)	0.061
Model 6	1.00 (Ref)	1.13 (0.87, 1.47)	1.05 (0.82, 1.34)	1.20 (0.92, 1.56)	1.15 (0.95, 1.39)	1.03 (0.99, 1.08)	0.162
Model 7	1.00 (Ref)	1.06 (0.81, 1.40)	1.07 (0.83, 1.38)	1.18 (0.90, 1.56)	1.12 (0.91, 1.37)	1.03 (0.98, 1.08)	0.267

*Model 1, crude model without adjustment; Model 2, adjusted for age, sex, educational level, marital status, annual family income; Model 3, adjusted for Model 2 plus smoking status, alcohol consumption, physical activity, DASH score, BMI, total energy intake, hypertension, type 2 diabetes mellitus and dyslipidemia status; Model 4, adjusted for Model 2 minus sex; Model 5, adjusted for Model 3 minus sex; Model 6, adjusted for Model 2 minus age; Model 7, adjusted for Model 3 minus age*.

Similar significant positive associations between the degree of pungency in spicy food and hyperuricemia were presented in Table 3. After adjusting for multiple variables, compared with participants who consumed less hot spicy food, the ORs (95% CIs) for those who ate moderately hot and very hot were 1.17 (1.05, 1.32) and 1.22 (0.92, 1.63), respectively. The corresponding OR (95% CI) for each level increment in the degree of pungency in spicy food was 1.15 (1.04, 1.26) (*P* trend = 0.004). Similarly, when exploring the relationship in the stratified analysis according to age and sex, a positive association was only found among men and participants aged 30–59, respectively (all *P* trend < 0.001). In addition, we found a significant interaction of age, sex, and spicy pungency on the risk of hyperuricemia (both *P*_interaction_ < 0.05). More stratified analyses were performed according to other demographic characteristics, and the linear associations between frequency and degree of pungency in spicy food and hyperuricemia are shown in Supplementary Table S1.

**Table 3:** Multivariable-adjusted associations (ORs and 95% CIs) between degree of pungency in spicy food consumption and risk of hyperuricemia (*N* = 16,843).

	Degree of pungency in spicy food^*^			**Each level increment**	***P* trend**
	**Low (*n* = 13,636)**	**Moderate (*n* = 2,819)**	**High (*n* = 388)**		
**Total**					
No. events (%)	1,992 (14.61)	565 (20.04)	71 (18.30)		
Model 1	1.00 (Ref)	1.47 (1.32, 1.63)	1.31 (1.01, 1.70)	1.33 (1.22, 1.44)	<0.001
Model 2	1.00 (Ref)	1.25 (1.12, 1.39)	1.20 (0.92, 1.57)	1.19 (1.09, 1.29)	<0.001
Model 3	1.00 (Ref)	1.17 (1.05, 1.32)	1.22 (0.92, 1.63)	1.15 (1.04, 1.26)	0.004
**Males**					
No. events (%)	1,273 (20.92)	461 (27.20)	52 (23.64)		
Model 1	1.00 (Ref)	1.41 (1.25, 1.60)	1.17 (0.85, 1.61)	1.27 (1.15, 1.41)	<0.001
Model 4	1.00 (Ref)	1.31 (1.16, 1.49)	1.31 (0.95, 1.81)	1.25 (1.12, 1.38)	<0.001
Model 5	1.00 (Ref)	1.26 (1.10, 1.44)	1.35 (0.96, 1.90)	1.22 (1.09, 1.36)	<0.001
**Females**					
No. events (%)	719 (9.52)	104 (9.25)	19 (11.31)		
Model 1	1.00 (Ref)	0.97 (0.78, 1.20)	1.21 (0.75, 1.97)	1.02 (0.86, 1.21)	0.797
Model 4	1.00 (Ref)	1.07 (0.86, 1.33)	1.21 (0.74, 1.98)	1.08 (0.92, 1.28)	0.351
Model 5	1.00 (Ref)	0.95 (0.75, 1.20)	1.11 (0.66, 1.88)	0.99 (0.83, 1.19)	0.947
**30–59 years old**					
No. events (%)	1,449 (13.91)	480 (20.70)	56 (21.54)		
Model 1	1.00 (Ref)	1.62 (1.44, 1.81)	1.70 (1.26, 2.30)	1.49 (1.36, 1.64)	<0.001
Model 6	1.00 (Ref)	1.31 (1.16, 1.47)	1.65 (1.20, 2.25)	1.30 (1.18, 1.43)	<0.001
Model 7	1.00 (Ref)	1.27 (1.11, 1.44)	1.62 (1.16, 2.28)	1.27 (1.14, 1.41)	<0.001
**60–79 years old**					
No. events (%)	543 (16.86)	85 (17.00)	15 (11.72)		
Model 1	1.00 (Ref)	1.01 (0.79, 1.30)	0.66 (0.38, 1.13)	0.91 (0.75, 1.09)	0.303
Model 6	1.00 (Ref)	1.01 (0.78, 1.30)	0.69 (0.40, 1.19)	0.92 (0.76, 1.11)	0.364
Model 7	1.00 (Ref)	0.93 (0.71, 1.22)	0.71 (0.40, 1.26)	0.89 (0.72, 1.08)	0.236

* *Among weekly spicy food consumers: participants who ate spicy food at least 1 day per week. Model 1, crude model without adjustment; Model 2, adjusted for age, sex, educational level, marital status, annual family income; Model 3, adjusted for Model 2 plus smoking status, alcohol consumption, physical activity, DASH score, BMI, total energy intake, hypertension, type 2 diabetes mellitus and dyslipidemia status; Model 4, adjusted for Model 2 minus sex; Model 5, adjusted for Model 3 minus sex; Model 6, adjusted for Model 2 minus age; Model 7, adjusted for Model 3 minus age*.

### Association of Spicy Food Intake With Serum Urate Level

Table 4 showed the associations of spicy food intake with serum urate level. Those participants who consumed spicy food more frequently were more likely to have higher serum urate levels. Compared with those who never ate spicy food, 3–5 d/week was associated with the highest serum urate level; the corresponding adjusted β (95% CI) was 5.21 (1.72, 8.7). A similar positive association was observed among men; the 3–5 d/week intake frequency was related to 10.37μmol/L (95% CI: 4.6, 16.14) higher serum urate level. However, there were no significant associations among women (*P* trend = 0.92). In the subgroup analysis stratified by age, we found similar positive associations among participants aged 30–59 and 60–79.

**Table 4:** Multivariable-adjusted associations (β coefficients and 95% CI) of frequency and degree of pungency in spicy food with serum urate level.

	**SUA (μmol/L)**	**Total**	**Males**	**Females**	**30–59 years old**	**60–79 years old**
**Frequency of spicy food intake**						
Never	307.23 ± 80.40	1.00 (Ref)	1.00 (Ref)	1.00 (Ref)	1.00 (Ref)	1.00 (Ref)
<1 d/week	307.56 ± 81.53	0.22 (−3.46, 3.90)	−0.38 (−6.59, 5.83)	0.67 (−3.61, 4.95)	−1.58 (−6.05, 2.89)	5.03 (−1.54, 11.60)
1–2 d/week	306.02 ± 81.49	−1.30 (−4.52, 1.92)	0.30 (−5.10, 5.71)	−2.45 (−6.20, 1.31)	−2.96 (−6.83, 0.92)	4.64 (−1.37, 10.64)
3–5 d/week	318.26 ± 86.01	5.21 (1.72, 8.70)	10.37 (4.60, 16.14)	0.86 (−3.27, 4.99)	4.45 (0.31, 8.59)	7.85 (1.06, 14.65)
6–7 d/week	319.09 ± 86.13	2.54 (−0.24, 5.32)	5.47 (0.86, 10.07)	−0.24 (−3.53, 3.05)	1.37 (−2.07, 4.82)	5.43 (0.65, 10.22)
*P* trend		0.006	0.001	0.920	0.026	0.038
Degree of pungency in spicy food^*^						
Low	311.94 ± 83.11	1.00 (Ref)	1.00 (Ref)	1.00 (Ref)	1.00 (Ref)	1.00 (Ref)
Moderate	334.39 ± 92.42	4.69 (1.93, 7.45)	7.19 (3.12, 11.26)	0.17 (−3.47, 3.80)	6.41 (3.40, 9.42)	−1.69 (−8.27, 4.88)
High	321.17 ± 86.37	−0.84 (−7.59, 5.91)	2.64 (−7.43, 12.70)	−2.24 (−11.02, 6.54)	3.97 (−4.12, 12.05)	−8.89 (−21.17, 3.39)
*P* trend		0.017	0.003	0.812	<0.001	0.183

* *Among weekly spicy food consumers: participants who ate spicy food at least 1 day per week. Adjusted model (except where it is the variable of interest): adjusted for age, sex, educational level, marital status, annual family income, smoking status, alcohol consumption, physical activity, DASH score, BMI, total energy intake, hypertension, type 2 diabetes mellitus and dyslipidemia status*.

In addition, compared with participants who ate less hot spicy food, those who consumed a stronger degree of pungency had higher serum urate levels. The corresponding adjusted β (95% CI) for moderately hot consumers was 4.69 (1.93, 7.45). Similarly, the moderate degree of pungency in spicy food intake was associated with 7.19μmol/L (95% CI: 3.12, 11.26) higher serum urate level among men, but no significant association was observed among women (*P* trend = 0.812). When further performed the multilevel-adjusted analysis stratified by age, a similar positive relationship was found among participants aged 30–59 (*P* trend <0.001) but not among those aged 60–79 (*P* trend = 0.183).

### Sensitivity Analyses

After excluding participants with self-reported peptic ulcer disease, coronary heart disease, stroke, and cancer, the results showed no marked change in the association of frequency of spicy food intake or degree of pungency in spicy food with the risk of hyperuricemia. Details were shown in Supplementary Tables S2, S3.

## Discussion

The current study explored the relationship between spicy food intake and hyperuricemia in Chinese adults. The frequency and robust pungency in spicy food consumption was positively associated with hyperuricemia. Those positive correlations were independent of socioeconomic and lifestyle factors frequently associated with spicy food consumption and hyperuricemia, such as age, sex, marital status, annual family income, educational level, smoking status, alcohol consumption, BMI, physical activity, DASH score, and total energy intake.

In line with previous studies, participants who consumed spicy food more frequently were more likely to be young, a man, current smokers, alcohol drinkers, and have higher BMI and total energy intake (36–39). We found that both frequency and degree of pungency in spicy food were positively associated with the prevalence of hyperuricemia as well as the serum uric acid level, which was consistent with the results of the Henan rural cohort study (29). Many previous studies can support our results. It seems that spicy food intake can lead to high BMI and obesity, which in turn to high serum uric acid and hyperuricemia (23, 40, 41). The findings of CKB reported that the spicy food strength and frequency might associate with increased BMI and other adiposity measures among half a million Chinese people (40). Consistent with the CKB study, a study conducted in Henan Province of China revealed that spicy flavor and spicy food frequency were associated with a higher risk of general obesity in Chinese rural populations (23). Additionally, a cross-sectional study suggested that spicy food intake might increase abdominal obesity risk by increasing energy intake (42). It is worth noting that many studies have demonstrated that obesity is an independent contributor to hyperuricemia in many countries, such as in United States, China, Japan, and Korea (41, 43–45). Furthermore, previous studies also found that spicy food consumption was associated with abnormal lipid metabolism among adults, and older people in China, respectively (24, 46). Abnormal lipid metabolism can lead to dyslipidemia, which was observed as an essential risk factor for hyperuricemia in many cross-sectional studies and longitudinal studies (41, 43, 45).

There is no exact mechanism research to prove that spicy foods increase the risk of hyperuricemia so far. Based on some previous studies, we speculated on the underlying causes of this relationship as follows. Firstly, the critical reason for this connection may be illustrated by the meat-dominated diet along with chili intake in Chinese cuisines. In Chinese cuisine, spicy foods are more meat than vegetables (47). Excessive intake of fatty meat along with spicy foods may increase the risk of obesity, which is known to be an independent risk factor for hyperuricemia (40). More importantly, meat contains more purines than other foods. In China, mutton, fish, and especially hotpot, which contains a lot of purine, are often flavored to taste spicy (40, 48). Excessive purine intake accompanied by spicy food can directly lead to high serum uric acid, and it may further develop into hyperuricemia as a result (48). Secondly, spicy food would increase carbohydrate intake and might result in high BMI and weight increase. And we know that fat accumulation may cause excessive uric acid production (44). Unlike the typical Western diet, which is meat-based, the Chinese take rice or noodles as their staple food. Because of improving the flavor, taste, color, and smell of food, spicy food was believed to enhance the palatability of food and also can be used to treat loss of appetite (16). Moreover, increasing carbohydrate intake was often used to relieve the burning sensation caused by spicy food (23). Thirdly, chili sauce and chili oil are widely used for flavoring in China, such as in hot pots and pickles, which may lead to excessive fat intake and further raise blood lipid levels (49). The increase in blood lipid levels, especially triglyceride, will induce more free fatty acids, accelerate the decomposition of adenosine triphosphate, and increase the production of uric acid (50). Furthermore, many studies have shown that consumers of spicy food with higher frequency or more robust pungency degrees were more likely to be drinkers (24, 37, 46, 51). Previous studies have found that alcohol drinking can promote uric acid synthesis and then block the excretion of uric acid, eventually leading to hyperuricemia (44, 52). In conclusion, excessive fat, carbohydrates, oil, and alcohol intake with spicy food may increase the risk of hyperuricemia. But after the total energy intake (protein, carbohydrate, and fat), DASH dietary pattern, and alcohol consumption have been adjusted in the current study, significant associations still were observed. In addition, positive relationships between spicy food and hyperuricemia were generally similar across subgroups stratified by alcohol consumption, more meat, and more fish, which to some extent showed the relationship was independent of these factors. Thus, the exact mechanism between spicy food and hyperuricemia remains unknown. This cross-sectional study only observed the association between spicy food intake and hyperuricemia but failed to establish a causal relationship. Further research is needed to validate this relationship.

Compared with those who never ate spicy food and consumed less hot, participants who consumed 3–5 days per week and were very hot had the highest risk of hyperuricemia. The results of non-linearity in the linkage between spicy food intake and the risk of hyperuricemia may be due to the extremely unbalance distribution of spicy food intake in our study. We further explored these relations in sex-stratified subgroups. Our results showed that men but not women participants who consumed higher frequency and a stronger degree of pungency in spicy food had significantly higher serum uric acid levels and a higher risk of hyperuricemia. This difference may be because participants who consumed spicy food frequently in our study were usually men. Moreover, the significant interaction between sex and spicy food intake further suggests that men may be more sensitive to the effect of spicy food intake on the risk of hyperuricemia. In addition, we should not ignore that anti-androgen therapy and female sex hormones could protect against the development of hyperuricemia (53, 54). When exploring the association in subgroup analysis stratified by age, significant positive associations were mainly found among 30–59 years old. This difference may be because youngers were more likely to eat spicy food in this study. More research is warranted to confirm the gender-dependent and age-dependent association and elucidate its mechanism.

Our study has several advantages as an observational study exploring the relationship between spicy food intake and hyperuricemia in Chongqing, China. Firstly, this research was carried out in Chongqing, China, a city known for its hot pot with a very high consumption of chili peppers. Secondly, the current study included a large sample size and adjusted for a broad range of confounding factors. It may be more representative to explore this association here, and the results of this study provide some insights into the association between spicy food consumption and hyperuricemia. In addition, not only according to the previous diagnosis results self-reported by participants, the determination of hyperuricemia but also based on the laboratory measurement results of their blood uric acid level. This strategy minimizes the omissions in patients with hyperuricemia.

Some limitations of this study should be noticed. Firstly, as a cross-sectional survey, we could not demonstrate a causal association and did not distinguish the effect of the spicy flavor by itself from other accompanying dietary factors, as we did not ask about the specific kinds of spicy food, such as spicy meat or vegetables. But confounding factors were adjusted, and subgroup analyses were performed to improve the study. Further prospective studies in large populations and clinical research are needed to verify the relationships. Secondly, a food frequency questionnaire was used to obtain the information on participants' spicy food intake, which might have reporting and recall bias, and no accurate data on the chili intake was obtained. Further research is needed to elucidate the specific mechanism of the effect of capsaicin on serum uric acid. Thirdly, all the individuals in the current study were voluntarily participating and were more likely to have high educational levels and healthy lifestyles, which may lead to underestimating the prevalence of various diseases. It is necessary to further verify the current results in other random sampling populations in future studies. Finally, this study only focused on Chongqing in the southwest region of China, which may not be representative of the whole Chinese population. More research in other areas is warranted to explore the association between spicy food and hyperuricemia.

## Conclusions

The present study demonstrated that frequency and degree of pungency in spicy food were positively associated with serum uric acid levels and the risk of hyperuricemia. In addition, this positive association was found significant in men and younger people but weaker in women and older people. Further prospective and intervention studies are warranted to verify the causal associations and elucidate specific mechanisms.

## Data Availability Statement

The raw data supporting the conclusions of this article will be made available by the authors, without undue reservation.

## Ethics Statement

This study was approved by the Ethics Committee of Sichuan University (No. K2016038), and all participants recruited in this study have signed informed consent.

## Author Contributions

QL analyzed the data and drafted the initial manuscript. RD, LC, XB, MX, YW, and JX were involved in the investigation, literature search, data acquisition, data analysis, and critically revising of the manuscript for important intellectual content. XL, WT, and JQ were involved in the conception, design, and data acquisition. XD and XT were involved in the manuscript's conception and design, data acquisition, and editing. All authors agree to be accountable for the content of the work.

## Funding

This work was supported by the National Key Research and Development Program of China (Grant Number: 2017YFC0907303), the Medical Scientific Research Project of Chongqing Health Commission (Grant Number: 2022WSJK021), and the Key Research and Development Project of the Science and Technology of Sichuan Province (Grant Number: 2020YFS0216).

## Conflict of Interest

The authors declare that the research was conducted in the absence of any commercial or financial relationships that could be construed as a potential conflict of interest.

## Publisher's Note

All claims expressed in this article are solely those of the authors and do not necessarily represent those of their affiliated organizations, or those of the publisher, the editors and the reviewers. Any product that may be evaluated in this article, or claim that may be made by its manufacturer, is not guaranteed or endorsed by the publisher.

## Abbreviations

CMEC: China Multi-Ethnic Cohort
CKB: China Kadoorie Biobank
BMI: Body mass index
METs: metabolic equivalent tasks
FFQ: Food Frequency Questionnaire
DASH: Dietary Approaches to Stop Hypertension
SBP: Systolic Blood Pressure
DBP: Diastolic Blood Pressure
FBG: Fasting Blood Glucose
TC: Total Cholesterol
TG: Triglycerides
LDLC: Low-Density Lipoprotein Cholesterol
HDLC: High-Density Lipoprotein Cholesterol
SD: Standard deviation
ANOVA: Analysis of Variance
OR: Odds ratio
CI: Confidence interval.
