# Supplementary material for: The Association Between Spicy Food Intake and Risk of Hyperuricemia Among Chinese Adults
Source: Front Public Health. 2022 Jul 6;10:919347. doi: 10.3389/fpubh.2022.919347 (PMC9298505; doi:10.3389/fpubh.2022.919347)
Supplement: Supplementary file 1 [file Table_1.pdf]

Table S1. Subgroup analyses of adjusted ORs (95% CIs) for hyperuricemia per level increment in the level of frequency and degree of pungency in spicy food consumption by demographic characteristics.

Subgroup	Frequency of spicy food intake (N=22 125)				Degree of pungency in spicy food (N=16 843)			
	Total	No.Events (%)	OR(95%CI)*	*P* value for interaction	Total	No.Events (%)	OR(95%CI)*	*P* value for interaction
Age(years old)				0.133				**<0.001**
30-59	16 320	2 381(14.6)	1.05(1.02,1.09)		12 994	1 985(15.3)	1.27(1.14,1.41)	
60-79	5 805	937(16.1)	1.03(0.98,1.08)		3 849	643(16.7)	0.89(0.72,1.08)	
Sex				**0.004**				**0.016**
Female	11 813	1 144(9.7)	1.01(0.97,1.06)		8 843	842(9.5)	0.99(0.83,1.19)	
Male	10 312	2 174(21.1)	1.06(1.02,1.10)		8 000	1 786(22.3)	1.22(1.09,1.36)	
Educational level				**0.046**				**0.019**
Illiteracy/primary school	7 194	918(12.8)	1.03(0.98,1.08)		4 908	637(13.0)	0.96(0.79,1.16)	
Junior high school	7 131	1 071(15.0)	1.04(0.98,1.09)		5 534	869(15.7)	1.22(1.03,1.44)	
High school and above	7 800	1 329(17.0)	1.06(1.01,1.12)		6 401	1 122(17.5)	1.22(1.06,1.41)	
Marital status				0.758				0.384
Married or cohabiting	19 451	2 888(14.9)	1.04(1.01,1.07)		14 916	2 302(15.4)	1.16(1.05,1.28)	
Separated/divorced/widowed/never married	2 674	430(16.1)	1.06(0.98,1.15)		1 927	326(16.9)	1.09(0.84,1.43)	
Annual family income (yuan)				0.484				0.607
<12000	2 452	309(12.6)	0.98(0.91,1.07)		1 540	203(13.2)	1.20(0.89,1.62)	
12000-19999	2 815	384(13.6)	1.13(1.04,1.22)		1 948	289(14.8)	1.05(0.77,1.43)	
20000-59999	7 629	1 060(13.9)	0.99(0.95,1.04)		5 824	810(13.9)	1.29(1.09,1.52)	
60000-99999	4 695	775(16.5)	1.12(1.05,1.20)		3 777	656(17.4)	1.04(0.85,1.26)	
>100000	4 534	790(17.4)	1.02(0.95,1.08)		3 754	670(17.8)	1.10(0.91,1.32)	
BMI (kg/m^2)				0.404				0.143
<24	9 772	814(8.3)	1.06(1.01,1.12)		7 346	633(8.6)	1.27(1.07,1.51)	

24-27.9	9 110	1 541(16.9)	1.04(1.00,1.09)		6 951	1 224(17.6)	1.12(0.98,1.29)	
⩾28	3 243	963(29.7)	1.01(0.95,1.07)		2 546	771(30.3)	1.11(0.92,1.33)	
Smoking status				**0.002**				0.070
Nonsmoker	16 223	2 112(13.0)	1.01(0.98,1.05)		11 918	1 566(13.1)	1.04(0.91,1.19)	
Current smoker	4 534	908(20.0)	1.09(1.02,1.16)		3 855	812(21.1)	1.30(1.12,1.50)	
Ex-smoker	1 368	298(21.8)	1.15(1.04,1.28)		1 070	250(23.4)	1.16(0.85,1.59)	
Drinking status [a]				0.470				0.407
No	19 382	2 671(13.8)	1.03(1.00,1.07)		14 431	2 050(14.2)	1.13(1.01,1.26)	
Yes	2 743	647(23.6)	1.06(0.97,1.15)		2 412	578(24.0)	1.24(1.03,1.48)	
Physical activity				0.737				0.952
Low	7 383	1 205(16.3)	1.05(1.00,1.10)		5 653	964(17.1)	1.20(1.03,1.40)	
Middle	7 367	1 182(16.0)	1.02(0.97,1.08)		5 575	930(16.7)	1.03(0.87,1.21)	
High	7 375	931(12.6)	1.04(0.99,1.10)		5 615	734(13.1)	1.24(1.04,1.48)	
More meat [b]				0.453				0.062
No	10 791	1 506(14.0)	1.03(0.99,1.07)		7 883	1 124(14.3)	1.05(0.90,1.22)	
Yes	11 334	1 812(16.0)	1.05(1.01,1.09)		8 960	1 504(16.8)	1.22(1.09,1.38)	
More fish [c]				0.910				0.865
No	17 071	2 490(14.6)	1.04(1.00,1.07)		12 678	1 919(15.1)	1.15(1.03,1.28)	
Yes	5 054	828(16.4)	1.04(0.97,1.11)		4 165	709(17.0)	1.14(0.95,1.37)	

[*] Adjusted model (except where it is the variable of interest): adjusted for age, sex, educational level, marital status, annual family income, smoking status, alcohol consumption, physical activity, DASH score, BMI, total energy intake, hypertension, type 2 diabetes mellitus and dyslipidemia status. [a] Alcohol drinking was defined as having more than 2 times of standard drinking (about one bottle of beer, 14 grams of alcohol)/week. [b] More meat and [c] more fish was defined as eating more than 500 grams per week, which was in line with the Chinese Dietary Guidelines (2022).
