# Supplementary material for: The Association Between Spicy Food Intake and Risk of Hyperuricemia Among Chinese Adults
Source: Front Public Health. 2022 Jul 6;10:919347. doi: 10.3389/fpubh.2022.919347 (PMC9298505; doi:10.3389/fpubh.2022.919347)
Supplement: Supplementary file 2 [file Table_2.pdf]

Table S2. Multivariable-adjusted associations (ORs and 95% CIs) between frequency of spicy food consumption and risk of hyperuricemia (N=20 133).

	Frequency of spicy food intake					Each level increment	*P* trend
	Never (n=2 702)	<1 d/week (n=1 973)	1-2 d/week (n=3 613)	3-5 d/week (n=2 624)	6-7 d/week (n=9 221)		
Total							
No. events(%)	347(12.8)	254(12.9)	481(13.3)	445(17.0)	1 460(15.8)		
Model 1	1.00(Ref)	1.00(0.84,1.19)	1.04(0.90,1.21)	1.39(1.19,1.61)	1.28(1.13,1.45)	1.08(1.05,1.11)	<0.001
Model 2	1.00(Ref)	1.00(0.84,1.20)	1.03(0.88,1.20)	1.31(1.12,1.54)	1.18(1.04,1.35)	1.05(1.02,1.08)	<0.001
Model 3	1.00(Ref)	0.97(0.80,1.17)	1.02(0.87,1.20)	1.28(1.08,1.51)	1.09(0.95,1.26)	1.03(1.00,1.06)	0.052
Males							
No. events(%)	203(16.4)	149(17.8)	298(19.8)	307(25.6)	1 043(22.6)		
Model 1	1.00(Ref)	1.11(0.88,1.40)	1.26(1.03,1.53)	1.75(1.44,2.14)	1.49(1.26,1.75)	1.10(1.07,1.14)	<0.001
Model 4	1.00(Ref)	0.98(0.78,1.24)	1.05(0.86,1.28)	1.41(1.15,1.72)	1.25(1.06,1.48)	1.07(1.03,1.11)	<0.001
Model 5	1.00(Ref)	0.98(0.76,1.26)	1.04(0.84,1.29)	1.41(1.13,1.75)	1.18(0.98,1.43)	1.05(1.01,1.10)	0.011
Females							
No. events(%)	144(9.8)	105(9.2)	183(8.7)	138(9.7)	417(9.1)		
Model 1	1.00(Ref)	0.93(0.72,1.21)	0.87(0.69,1.10)	0.98(0.77,1.26)	0.91(0.75,1.12)	0.99(0.94,1.03)	0.584
Model 4	1.00(Ref)	1.07(0.82,1.40)	1.06(0.84,1.34)	1.21(0.94,1.55)	1.10(0.89,1.35)	1.02(0.98,1.07)	0.351
Model 5	1.00(Ref)	0.97(0.73,1.29)	1.02(0.80,1.31)	1.10(0.84,1.44)	0.98(0.79,1.21)	1.00(0.95,1.05)	0.890
30-59 years old							
No. events(%)	189(11.6)	174(11.9)	368(12.6)	358(16.8)	1 146(15.8)		
Model 1	1.00(Ref)	1.03(0.83,1.28)	1.11(0.92,1.33)	1.55(1.28,1.87)	1.43(1.22,1.69)	1.11(1.07,1.15)	<0.001
Model 6	1.00(Ref)	1.03(0.82,1.29)	1.08(0.89,1.31)	1.42(1.17,1.73)	1.27(1.08,1.51)	1.07(1.03,1.11)	<0.001
Model 7	1.00(Ref)	1.05(0.82,1.33)	1.12(0.91,1.38)	1.48(1.20,1.83)	1.22(1.02,1.47)	1.05(1.01,1.09)	0.008
60-79 years old							
No. events(%)	158(14.8)	80(15.8)	113(16.2)	87(17.7)	314(16.1)		

Model 1	1.00(Ref)	1.08(0.80,1.44)	1.11(0.85,1.44)	1.24(0.93,1.64)	1.10(0.90,1.36)	1.02(0.98,1.08)	0.338
Model 6	1.00(Ref)	1.06(0.79,1.42)	1.06(0.81,1.38)	1.16(0.87,1.55)	1.07(0.87,1.32)	1.02(0.97,1.07)	0.512
Model 7	1.00(Ref)	0.98(0.72,1.34)	1.02(0.77,1.35)	1.08(0.80,1.47)	1.01(0.81,1.26)	1.01(0.95,1.06)	0.864

Model 1: crude model without adjustment; Model 2: adjusted for age, sex, educational level, marital status, annual family income; Model 3: adjusted for Modle 2 plus smoking status, alcohol consumption, physical activity, DASH score, BMI, total energy intake, hypertension, type 2 diabetes mellitus and dyslipidemia status; Model 4: adjusted for Model 2 minus sex; Model 5: adjusted for Model 3 minus sex; Model 6: adjusted for Model 2 minus age; Model 7: adjusted for Model 3 minus age.
