# Supplementary material for: The Association Between Spicy Food Intake and Risk of Hyperuricemia Among Chinese Adults
Source: Front Public Health. 2022 Jul 6;10:919347. doi: 10.3389/fpubh.2022.919347 (PMC9298505; doi:10.3389/fpubh.2022.919347)
Supplement: Supplementary file 3 [file Table_3.pdf]

Table S3. Multivariable-adjusted associations (ORs and 95% CIs) between degree of pungency in spicy food consumption and risk of hyperuricemia (N=15 458).

	Degree of pungency in spicy food *			Each level increment	*P* trend
	Low (n=12 486)	Moderate (n=2 621)	High (n=351)		
Total					
No. events(%)	1 802(14.4)	519(19.8)	65(18.5)		
Model 1	1.00(Ref)	1.46(1.31,1.63)	1.35,1.03,1.77)	1.33(1.22,1.46)	<0.001
Model 2	1.00(Ref)	1.24(1.11,1.38)	1.26(0.95,1.67)	1.19(1.09,1.30)	<0.001
Model 3	1.00(Ref)	1.18(1.05,1.33)	1.32(0.98,1.78)	1.17(1.06,1.29)	0.002
Males					
No. events(%)	1 176(21.1)	424(27.2)	48(24.7)		
Model 1	1.00(Ref)	1.40(1.23,1.59)	1.23(0.88,1.72)	1.28(1.15,1.42)	<0.001
Model 4	1.00(Ref)	1.30(1.14,1.49)	1.37(0.98,1.92)	1.25(1.13,1.39)	<0.001
Model 5	1.00(Ref)	1.27(1.10,1.46)	1.46(1.02,2.09)	1.24(1.11,1.40)	<0.001
Females					
No. events(%)	626(9.1)	95(8.9)	17(10.8)		
Model 1	1.00(Ref)	0.99(0.79,1.24)	1.22(0.73,2.03)	1.03(0.87,1.23)	0.710
Model 4	1.00(Ref)	1.06(0.84,1.34)	1.22(0.73,2.04)	1.08(0.90,1.29)	0.400
Model 5	1.00(Ref)	0.95(0.74,1.21)	1.15(0.66,1.99)	1.00(0.83,1.21)	0.991
30-59 years old					
No. events(%)	1 369(13.9)	449(20.4)	54(21.7)		
Model 1	1.00(Ref)	1.60(1.42,1.80)	1.72(1.27,2.34)	1.48(1.35,1.63)	<0.001
Model 6	1.00(Ref)	1.30(1.15,1.47)	1.67(1.22,2.30)	1.30(1.17,1.43)	<0.001
Model 7	1.00(Ref)	1.25(1.10,1.43)	1.68(1.19,2.37)	1.27(1.14,1.42)	<0.001
60-79 years old					
No. events(%)	433(16.6)	70(16.5)	11(10.8)		

Model 1	1.00(Ref)	0.99(0.75,1.31)	0.61(0.32,1.15)	0.89(0.72,1.10)	0.268
Model 6	1.00(Ref)	0.98(0.74,1.30)	0.63(0.33,1.19)	0.89(0.72,1.10)	0.282
Model 7	1.00(Ref)	0.96(0.72,1.30)	0.71(0.37,1.37)	0.91(0.72,1.13)	0.384

*among weekly spicy food consumers: participants who ate spicy food at least one day per week. Model 1: crude model without adjustment; Model 2: adjusted for age, sex, educational level, marital status, annual family income; Model 3: adjusted for Modle 2 plus smoking status, alcohol consumption, physical activity, DASH score, BMI, total energy intake, hypertension, type 2 diabetes mellitus and dyslipidemia status; Model 4: adjusted for Model 2 minus sex; Model 5: adjusted for Model 3 minus sex; Model 6: adjusted for Model 2 minus age; Model 7: adjusted for Model 3 minus age.
